# Comparison of owner-reported behavioral characteristics among genetically clustered breeds of dog (*Canis familiaris*).

**DOI:** 10.1038/srep17710

**Published:** 2015-12-18

**Authors:** Akiko Tonoike, Miho Nagasawa, Kazutaka Mogi, James A. Serpell, Hisashi Ohtsuki, Takefumi Kikusui

**Affiliations:** 1 Department of Animal Science and Biotechnology, Azabu University, Sagamihara, Kanagawa, Japan; 2 Department of Physiology, Jichi Medical University, Shimotsuke, Tochigi, Japan; 3 School of Veterinary Medicine, University of Pennsylvania, Philadelphia, Pennsylvania, USA; 4 Department of Evolutionary Studies of Biosystems, School of Advanced Sciences, The Graduate University for Advanced Studies (SOKENDAI).

## Abstract

During the domestication process, dogs were selected for their suitability for multiple purposes, resulting in a variety of behavioral characteristics. In particular, the ancient group of breeds that is genetically closer to wolves may show different behavioral characteristics when compared to other breed groups. Here, we used questionnaire evaluations of dog behavior to investigate whether behavioral characteristics of dogs were different among genetically clustered breed groups. A standardized questionnaire, the Canine Behavioral Assessment and Research Questionnaire (C-BARQ), was used, and breed group differences of privately-owned dogs from Japan (n = 2,951) and the United States (n = 10,389) were analyzed. Results indicated that dogs in the ancient and spitz breed group showed low attachment and attention-seeking behavior. This characteristic distinguished the ancient group from any other breed groups with presumed modern European origins, and may therefore, be an ancestral trait.

The dog *(Canis familiaris)* was the first animal to be domesticated^1^ and today hundreds of different breeds are recognized. Breeds seem to be different in several aspects of their behavior due to the effects of artificial selection^2^,^3^,^4^,^5^. Although breeds are traditionally classified by the jobs they were originally selected to perform, parallel selection for other traits, such as suitability as pets, has also affected modern breed-typical behavior^6^. With the remarkable improvement of technologies available for genetic analysis, genetic relationships in dog breeds have recently been studied and genetic classifications of dog breeds have been constructed^7^,^8^. As a result, although dog breeds have traditionally been classified by their roles in human activities, historical records, and physical phenotypes, it is now possible to classify them based on patterns of genetic variation^9^,^10^,^11^.

Cladogram analysis of dog genes showed the separation of several breeds with supposedly ancient origins from a large group of breeds with presumed modern European origins^7^,^8^. Modern European breeds are the products of controlled breeding practices since the Victorian era, and because they have originated recently and lack deep histories, the genetic groups have short internodes and low bootstrap support. On the other hand, ancient breeds are highly divergent and are distinct from modern European breeds. Since the dogs from these ancient breeds are genetically related most closely to wolves, they may exhibit remnants of wolves’ behavioral, morphological and physiological characteristics.

The Canine Behavioral Assessment and Research Questionnaire (C-BARQ) is designed to provide dog owners and professionals with standardized evaluations of canine temperament and behavior^12^. The C-BARQ has also been translated for use in Japan^13^,^14^ after examination of the validity of questionnaire items^15^. In this study, we used C-BARQ evaluations of dogs to investigate whether the behavioral characteristics of dogs are different among genetically clustered breed groups. Although C-BARQ scores are obtained from dog owners and may therefore be influenced by subjective biases, the use of this instrument allows the standardized assessment of behavior in very large numbers of dogs, and has proven useful for studying breed differences in behavior^16^,^17^,^18^. Several studies comparing wolves, dogs, and other canids, suggest that behavioral changes were critical during the early stages of the domestication process^19^,^20^,^21^. We investigated the behavioral characteristics of breeds, especially those belonging to the ancient group, to understand the characteristics of this highly divergent group of ancient breeds.

## Materials and Methods

### Questionnaire

Behavioral data in the present study were collected from dog owners using the C-BARQ, which included 100 questions that asked owners to indicate how their dogs have responded in the recent past to a variety of common events and stimuli using a series of 0–4 rating scales. The C-BARQ is a standardized questionnaire that is widely used to assess the prevalence and severity of behavioral problems in dogs. The various C-BARQ item and subscale scores have also been shown to provide an accurate measure of canine behavioral phenotypes. Seven of the original 11 subscales were validated using a panel of 200 dogs previously diagnosed with specific behavior problems^12^. More recently, other studies have provided criterion validation of the C-BARQ by demonstrating associations between factor and item scores and training outcomes in working dogs^22^, the performance of dogs in various standardized behavioral tests^23^,^24^,^25^,^26^, and neurophysiological markers of canine anxiety and compulsive disorders^27^,^28^. The original C-BARQ was translated into Japanese by two behavioral professionals and reviewed by two professors^15^. Twenty-two out of 100 questions were eliminated due to the cultural and environmental differences between Japan and the USA, resulting in 78 questions for the Japanese version.

C-BARQ data were collected via the freely accessible websites http://www.cbarq.org (US, from April 2006) and http://cbarq.inutokurasu.jp/(JPN, from September 2010). Before answering the questionnaire, dog owners were asked to provide information about their dogs, such as its breed, age, sex, neuter status, body weight, age when acquired, where acquired, and the presence of any health problems. The online survey was advertised via articles in newspapers, magazines, online news, etc., in each country. The C-BARQ database was used for different purpose 29. The Ethical committee of Azabu University approved this study. We obtained the informed consent from all respondents and our methods were carried out in accordance with the approved guidelines.

### Statistical analyses

Data from the completed questionnaires were subjected to factor analysis. Parallel analysis was used to determine the number of interpretable factors that could be extracted, and varimax rotation was used to identify empirical groupings of items that measured different behavioral traits. The Cronbach’s α coefficient was calculated to assess internal consistency (reliability) of extracted factors; this coefficient describes how well a group of questionnaire items focuses on a single idea or construct. For comparison of the factors, we calculated the average of item scores composing each factor, which was analyzed as a factor score. The factor scores were then analyzed using generalized linear models These were analyzed by SPSS v.19.0 (SPSS Japan Inc., IBM company), except for the parallel analysis (R v. 3.0.0, 2013-04-03, The R Foundation for Statistical Computing).

## Results

### Subjects

A total of 5,377 C-BARQ questionnaires were completed in Japan. Dogs that were <1 or >7 years of age or had severe or chronic health problems were excluded, leaving a total of 3,098 completed questionnaires (57.76%) that were considered valid. The age cut-off was chosen to eliminate dogs whose behavior might have been affected by immaturity or senescence (in the case of some large or giant breeds). The response rates for each of the 78 questions in the questionnaire ranged from 39.22% to 99.86% (median, 98.39%, mode, 99.15%). The low response rates obtained for some questionnaire items were primarily due to the fact that the questionnaire’s focus on events and stimuli occurring in recent past tended to exclude uncommon events and situations. Among the 14,481 questionnaires completed in the United States, 10,500 satisfied the requirements above (72.51%). The response rates for each of the 100 questions in the questionnaire ranged from 81.57% to 99.72% (median, 97.85%, mode, 98.04%). Fifteen questions with response rates <85.0% either in Japanese or US data were excluded for further analyses. Any questionnaires that had <75.0% response rates were also excluded, leaving 2,951 (54.88%) and 10,389 (71.74%) completed questionnaires that could be used in analyses in Japan and the United States, respectively.

### Factor analysis

For the factor analysis we selected 59 breeds that were common to both countries and then matched the samples for sex and number of dog for each country in order to eliminate any sex or country biases (n = 1,252 each, Supplementary Table 1). Sixty-three of the questionnaire items common to both countries were analyzed by factor analysis and parallel analysis, and these items were sorted into 12 factors. After removing the items with factor loadings of <0.4, the remaining items were analyzed by factor analysis again, and yielded 12 factors. After removing the items with factor loadings of <0.4 again, the remaining items were analyzed by factor analysis, and again yielded 12 factors that accounted for 54.96% of the common variance in item scores. Out of these twelve factors, eleven were found to have adequate Cronbach’s α values (≥0.7) (Table 1). The following eleven factors were extracted: aggression to unfamiliar persons (F1), fear of unfamiliar persons (F2), trainability (F3), separation-related behavior (F4), energy and restlessness(F5), fear of non-social stimuli (F6), aggression to household members (F7), fear of unfamiliar dogs (F8), aggression to unfamiliar dogs (F9), attachment and attention-seeking (F11), and aggression to persons passing near the house (F12). These results are shown in Table 1.

### The influence of breeds on C-BARQ factor scores

Using the generalized linear model, the influence of breeds and various demographic variables on C-BARQ factor scores were examined. The dog breeds were separated into eight breed groups according to the cladogram suggested by vonHoldt (2010). The eight groups consist of 1) ancient and spitz breeds, 2) toy dogs, 3) spaniels, scent hounds, and poodles, 4) working dogs, 5) small terriers, 6) sight hounds and herding dogs, 7) retrievers, and 8) mastiff-like dogs. As the Shiba Inu breed was not included in vonHoldt’s cladogram, we classified them into the ancient and spitz breed group according to the cladogram suggested by Parker (2004). Other dog breeds not shown in vonHoldt’s cladogram were eliminated from further analyses. The dog breeds in each breed group are shown in Table 2.

**Figure 1:**
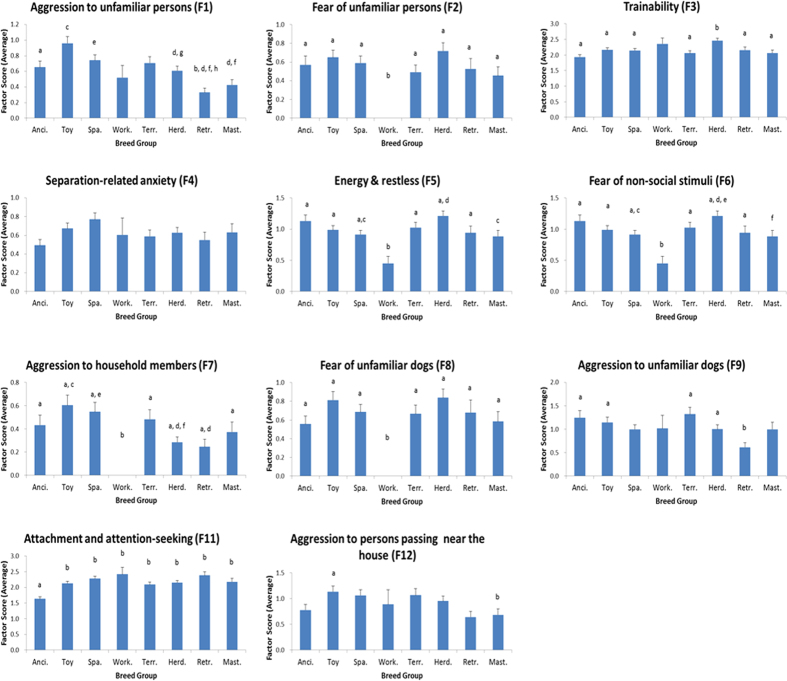
Average factor scores for breed groups. The dog breeds were separated into eight breed groups according to the cladogram, Ancient and spitz breeds: 1, Toy dogs: 2, Spaniels, scent hounds, and poodles: 3, Working dogs: 4, Small terriers: 5, Sight hounds and herding dogs: 6, Retrievers: 7, Mastiff-like dogs: 8. a vs b, p < 0.05; c vs d, p < 0.05; e vs f, p < 0.05; g vs h, p < 0.05.

We analyzed the relationships between breed groups and C-BARQ factor scores while taking into account the possible intervening effects of the following 8 variables: country, sex, spay/neuter status, source from which dogs were acquired, owner’s experience of dog-ownership, dog’s age when acquired, dog’s body weight, dog’s age at the time of evaluation. These variables have previously been shown to influence the expression of behavior in dogs^16^,^30^,^31^,^32^. All of the factor scores were explained significantly by the variables, although most of the variance was explained by breed groups, country, sex, spay/neuter status, and source from which dogs were acquired. Significant interactions between breed group and other variables were also found. Results are shown in Table 3 and Fig. 1.

Toy dogs obtained the highest scores for Factor 1 (aggression to unfamiliar persons), and were significantly more aggressive in this context than sight hounds and herding dogs, retrievers, and mastiff-like dogs. Spaniels, scent hounds, and poodles were significantly more aggressive to unfamiliar persons than retrievers and mastiff-like dogs. The ancient and spitz breed group and sight hounds and herding dogs were significantly more aggressive to unfamiliar persons than retrievers. For F2 (fear of unfamiliar persons), working dogs obtained the lowest scores, and were significantly lower than all other breed groups. For F3 (trainability), sight hounds and herding dogs obtained the highest scores, and were significantly more trainable than all other breed groups except working dogs. For F4 (separation-related anxiety), there were no breed group differences. For F5 (energy and restlessness), working dogs obtained the lowest scores, and were significantly lower than all other breed groups except mastiff-like dogs. Sight hounds and herding dogs obtained the highest scores, and were significantly higher than the spaniels, scent hounds, and poodles breed group and mastiff-like dogs. For F6 (fear of non-social stimuli), sight hounds and herding dogs obtained the highest scores, and were significantly higher than the spaniels, scent hounds, and poodles breed group and mastiff-like dogs. Working dogs obtained the lowest scores, and were significantly lower than all other breed groups except mastiff-like dogs. For F7 (aggression to household members), working dogs obtained the lowest scores, and were significantly less aggressive in this context than all other breed groups. Toy dogs obtained the highest scores, and were significantly more aggressive to household members than the sight hounds and herding dogs breed group and retrievers. The spaniels, scent hounds, and poodles breed group was significantly more aggressive to household members than the sight hounds and herding dogs breed group. For F8 (fear of unfamiliar dogs), working dogs obtained the lowest score, and were significantly lower than all other breed groups. For F9 (aggression to unfamiliar dogs), retrievers obtained the lowest scores, and were significantly less aggressive in this context than ancient and spitz breeds, toy dogs, the small terriers, and the sight hounds and herding dogs breed group. For F11 (attachment and attention-seeking), the ancient and spitz breed group obtained the lowest scores, and were significantly lower than all other breed groups. For F12 (aggression to persons passing near the house), toy dogs obtained the highest scores, and were significantly more aggressive in this context than mastiff-like dogs.

Some factors were different between US and Japan (US > JPN; F2, F3, F4, F8, F11 JPN > US; F5, F6), and between male and female (male > female; F9 female > male; F2, F8). Some factors were affected by spay/neuter status (intact > neutered; F4 neutered > intact; F5, F6), source from which dogs were acquired (F1; friend or relative > breeder, shelter > breeder F3; breeder > pet store, other > pet store) and owner’s experience of dog-ownership (first ownership > second and more ownership; F4 second and more ownership > first ownership; F3). Body weight, dog’s age at the time of evaluation and dog’s age when acquired also influenced some factors (body weight; F2, F4, F5, F6, F8, F11 dog’s age at evaluation; F3, F4, F9, F11 dog’s age when acquired; F1, F3, F5, F6, F12). The environment for dogs and their owners are different in Japan and US. For example, pet stores are a popular source of dog acquisition in Japan compared with the US where most purebred dogs are acquired directly from breeders. In order to investigate the effect of country on breed group differences, we separated the questionnaire data into two groups -dogs living in Japan and the USA- and analyzed for the breed group differences separately in each group. There were some differences between countries, the primary breed group differences remained the same in both countries, especially with respect to F11 (attachment and attention-seeking), even though there were large differences in the environment surrounding the dogs in two countries. The results are shown in Supplemental Table 2.

Additionally, we conducted cluster analyses of the factors using breed medians and Ward’s method. All of the breeds of the ancient and spitz breed group are clustered in one group in the dendrogram branches associated with F2 (fear of unfamiliar persons), F4 (separation-related anxiety) and F11 (attachment and attention-seeking). Four out of five breeds of the ancient and spitz breed group are also clustered in one group in the branch associated with F8 (fear of unfamiliar dogs). In F11 (attachment and attention-seeking), two clusters were identified and all of five breeds of ancient and spitz breed group were classified into the same cluster. Five other breeds also clustered as showing low levels of attachment and attention-seeking, including two terriers (West Highland White Terrier, Cairn Terrier), two sight hounds (Whippet, Borzoi), and the Great Dane. In F4 (separation-related anxiety), two clusters were identified and all of five breeds of ancient and spitz breed group were classified into the same cluster. Fifteen other breeds are also clustered as showing low levels of separation anxiety. In F2 (fear of unfamiliar persons), two clusters were identified and eleven breeds (Chihuahua, Poodle (Toy), Boxer, Yorkshire Terrier, Shetland Sheepdog, Whippet, Maltese, Miniature Pinscher, Great Dane, Cocker Spaniel (American), and Italian Greyhound) were in one cluster and all other breeds were in the other. The trees for F2, F4 and F11 are shown in Supplementary Figure 1, 2 and 3.

## Discussion

Using a validated online behavioral evaluation system (C-BARQ), we collected data on the behavioral characteristics of dogs in Japan and the United States, and investigated differences among genetically classified breed groups. Overall, most of the variance in C-BARQ factor scores was explained by the variables; breed group, country, sex, spay/neuter status, and source from which dogs were acquired. Significant interactions between breed group and other variables were also found, indicating that the behaviors evaluated by C-BARQ were influenced by genetic origins, hormonal status and environmental factors, such as country. Some factors were clearly explained by breed group differences. These differences may be related to the effects of direct selection for behavioral characteristics or due to differences in the conditions of life that different breeds experience during development. Since it is hard to believe that all ancient breeds grew up in similar environments that were distinct from those of all modern breeds, it appears unlikely that the observed breed group differences are due solely to environmental factors. Furthermore, when we separated the questionnaire data into two groups—dogs living in Japan and the United States—and analyzed for breed group differences separately in each group (Supplementary Table 2), we identified similar breed group differences in behavior in both countries. This finding supports the view that these differences are primarily due to genetic factors. The most unique among the eight breed groups is the working dog group, which shows the lowest levels of fear of unfamiliar persons, non-social stimuli, and unfamiliar dogs, and the lowest scores for energy, hyperactivity, and aggression to household members. Working dogs are used as police dogs, military dogs, watch dogs, and may be under strong selection for these characteristics. Even those that live as family pets, may still retain the the effects of past selection for working roles. Since the data for working dogs are from only two breeds, Doberman Pinscher and German Shepherd, there is a possibility that the uniqueness of the working dog group is related to the small size of this group. Similarly, the trainability of sight hounds and herding dogs may be high because of direct selection for this characteristic. Most interestingly, the scores for attachment and attention-seeking of the ancient and spitz breeds group is different from the scores of any other breed group with presumed modern European origins. Some studies suggested that even hand-reared wolves did not show attachment-like behavior like dogs^21^,^33^; therefore, the unique characteristic of this breed group may be one of the remnants of wolves’ behavioral characteristics and may be very informative of understanding the dog domestication processes. “Attachment” in C-BARQ is defined by owners’ responses to questions concerning the dog’s tendency “to follow members of the household from room to room about the house,” “to sit close to or in contact with a member of the household when that individual is sitting down,” “to nudge, nuzzle, or paw a member of the household for attention when that individual is sitting down,” and “to become agitated when a member of the household shows affection for another person or animal.” Considering the history of these breeds, it seems unlikely that dogs in the ancient and spitz breed group were selected for low degrees of attachment and attention seeking. Rather, it is more likely that the capacity to form attachments for humans was an important component of the evolution of modern dogs. Furthermore, we believe that the cluster analysis of the attachment and attention-seeking traits, in which all five breeds in the ancient and spitz breeds groups clustered in a small tightly clustered group of 10 breeds, clearly separated from the 36 other breeds, supports our interpretation that low levels of attachment and attention-seeking are a distinctive behavioral characteristic of the ancient and spitz breed groups. The five other breeds that clustered with the ancient and spitz breeds for attachment and attention-seeking may have developed lower levels of attachment secondarily as adaptations for hunting independently of human guidance. We also investigated the influence of the two different grouping methods, of vonHoldt *et al.*^8^ and Parker *et al.*^11^ on the low attachment tendency in the ancient and spitz breed group. The low attachment tendency in the ancient and spitz breed group was stable for both grouping methods.

Although some of the breeds in the ancient and spitz group have practical functions such as pulling sleds (e.g. Siberian husky), their C-BARQ scores for attachment and attention-seeking are not different from the other breeds in the ancient and spitz group. This may be because they are primarily motivated to run in groups without formal training or the need to attend to or follow instructions from a human handler^1^. We also need to be careful about interpreting the close relationship of these breeds to wolves in the cladogram because there may be an influence of recent crossing with wolves.

Previous discussions of the behavioral changes associated with the domestication of the dog have tended to emphasize the role of selection for the trait of “tameness” (i.e. loss of fearful or aggressive responses toward humans)^34^,^35^. However, a previous study of species differences in behavior towards humans between hand-reared dogs and wolf pups also revealed that even wolves that have been intensively socialized do not show the same levels of attachment behavior towards humans that dogs do^33^. This suggests that in addition to tameness, dogs may acquired high levels of attachment and attention-seeking behavior toward humans during the domestication process. In a famous series of experiments involving farmed foxes (*Vulpes vulpes*), individuals with low aggressive-fearful reactions to humans were selectively bred for over forty generations. This led to a unique population of foxes that also gradually showed high attachment behaviors, such as actively seeking contact with humans, tail-wagging in anticipation of social contact, licking experimenters’ faces and hands, and following them like dogs^36^. Considering the results of our study, together with the results of such experiments, we believe that one of the earliest stages of dog domestication may have involved selection for not only low aggressive-fearful tendencies in ancestral wolves toward humans, but also the early development of human-directed attachment behavior.

Despite their low attachment and attention-seeking tendencies, the aggressive and fearful reactions towards humans were relatively low in the ancient and spitz breed group. It is possible that domestication may have involved a two-stage process, with selection for low aggressive and fearful tendencies occurring in the first stage, and selection for prosociality (attachment and attention-seeking) occurring later, perhaps in association with the development of more specialized working roles. As a result, the ancient and spitz breeds may retain the low aggressive and fearful tendencies associated with stage 1, but lack the strong prosocial traits associated with stage 2 and more modern breeds of dog. Viewed in this light, the aggressiveness toward humans characteristic of toy dogs may be a secondary development concomitant with their small body size which renders them less of a threat to humans. Or it may be that toy breeds are less adequately socialized by their owners. This association between small body size and aggression in dogs confirms the findings of previous studies^17^.

The findings of the present study, namely that the ancient and spitz breed group shows the lowest attachment levels and is significantly different from other breed groups, confirms the idea that selective processes may have taken place during domestication on genetic changes affecting the attachment system, and that the consistently low attachment levels found in this group of breeds may be a remnant of an earlier stage of dog evolution. Since we could not fully describe the contribution of environmental factors to these observed breed differences in behavior, future investigations will need to take into account the possible effects of breed-specific environmental influences.

## Additional Information

**How to cite this article**: Tonoike, A. *et al.* Comparison of owner-reported behavioral characteristics among genetically clustered breeds of dog (*Canis familiaris*). *Sci. Rep.* **5**, 17710; doi: 10.1038/srep17710 (2015).

## Supplementary Material

Supplementary Figure 1

Supplementary Figure 2

Supplementary Figure 3

Supplementary Table 1

Supplementary Table 2

Supplementary figure legends

## Figures and Tables

**Table 1:** Factor loading of questionnaire items constituting each factor.

Factors & questionnaire items	Factor loadings	SS loadings	Proportion Var	Cumulative Var	Cronbach’s α
***Aggression to unfamiliar persons***		5.13	0.10	0.10	0.92
When approached directly by an unfamiliar adult while being walked or exercised on a leash	0.836				
When approached directly by an unfamiliar child while being walked or exercised on a leash	0.744				
When an unfamiliar person approaches the owner or a member of the owner’s family at home	0.670				
When an unfamiliar person approaches the owner or a member of the owner’s family away from home	0.792				
When mailmen or other delivery workers approach the home	0.604				
When an unfamiliar person tries to touch or pet the dog	0.806				
Toward unfamiliar persons visiting the home	0.717				
***Fear of unfamiliar persons***		2.79	0.05	0.15	0.90
When approached directly by an unfamiliar adult while away from the home	0.823				
When approached directly by an unfamiliar child while away from the home	0.747				
When unfamiliar persons visit the home	0.689				
When an unfamiliar person tries to touch or pet the dog	0.761				
***Trainability***		2.77	0.05	0.20	0.81
Returns immediately when called while off leash	0.667				
Obeys a sit command immediately	0.676				
Obeys a stay command immediately	0.717				
Seems to attend to or listen closely to everything the owner says or does	0.693				
Not slow to respond to correction or punishment	0.619				
Not easitly distracted by interesting sights, sounds, or smell	0.447				
***Separation-related anxiety***		2.73	0.05	0.25	0.76
Excessive salivation when left or about to be left on its owner	0.497				
Whining when left or about to be left on its owner	0.714				
Barking when left or about to be left on its owner	0.735				
Howling when left or about to be left on its owner	0.608				
Chewing/scratching at doors, floor, windows, curtains, etc.	0.462				
Loss of appetite when left or about to be left on its owner	0.403				
***Energy and restless***		2.65	0.05	0.30	0.72
Not shaking, shivering, or trembling when left or about to be left on its owner	0.441				
Restlessness, agitation, or pacing when left or about to be left on its owner	0.477				
When a member of the household returns home after a brief absence	0.690				
When visitors arrive at its home	0.480				
Playful, puppyish, boisterous	0.694				
Active, energetic, always on the go	0.493				
***Fear of non-social stimuli***		2.27	0.04	0.34	0.75
In response to sudden or loud noises	0.665				
In response to strange or unfamiliar objects on or near the sidewalk	0.671				
During thunderstorms	0.422				
When first exposed to unfamiliar situations	0.468				
In response to wind or wind-blown objects	0.699				
***Aggression to household members***		2.25	0.04	0.38	0.84
When toys, bones, or other objects are taken away by a member of the household	0.577				
When approached directly by a member of the household while it is eating	0.773				
When food is taken away by a member of the household	0.849				
When a member of the household retrieves food or objects stolen by the dog	0.626				
***Fear of unfamiliar dogs***		2.21	0.04	0.42	0.88
When approached directly by an unfamiliar dog of the same or larger size	0.769				
When approached directly by an unfamiliar dog of a smaller size	0.792				
When barked, growled, or lunged at by unfamiliar dog	0.756				
***Aggression to unfamiliar dogs***		2.10	0.04	0.46	0.89
When approached directly by an unfamiliar male dog while being walked or exercised on a leash	0.849				
When approached directly by an unfamiliar female dog while being walked or exercised on a leash	0.840				
When barked, growled, or lunged at by unfamiliar dog	0.642				
***Attachment and attention-seeking***		1.78	0.03	0.53	0.70
Tends to follow a member of household from room to room about the house	0.541				
Tends to sit close to or in contact with a member of the household when that individual is sitting down	0.672				
Tends to nudge, nuzzle, or paw a member of the household for attention when that individual is sitting down	0.701				
Becomes agitated when a member of the household shows affection for another person	0.466				
***Aggression to persons passing near the house***		1.15	0.02	0.55	0.89
When strangers walk past the home while the dog is in the yard	0.652				
When joggers, cyclists, roller skaters, or skateboarders pass the home while the dog is in the yard	0.607				

**Table 2:** Genetically clustered breed groups used for statistical analysis.

Breed groups	Breeds
**Ancient and spitz breeds (152)**	Basenji(JPN4, US4), Shiba Inu(64, 64), Akita(4, 4), Siberian Husky(2, 2), Samoyed(2, 2)
**Toy dogs (612)**	Shih Tzu(40, 40), Chihuahua(110, 110), Pug(46, 46), Papillon(24, 24), Pomeranian(50, 50), Miniature Pinscher(26, 26), Brussels Griffon(2, 2), Pekingese(8, 8)
**Spaniels, scent hounds, and poodles (388)**	American Cocker Spaniel(14, 14), English Cocker Spaniel(10, 10), English Springer Spaniel(4, 4), Cavalier King Charles Spaniel(36, 36), Brittany(2, 2), Beagle(42, 42), Bichon Frise(10, 10), Maltese(32, 32), Toy Poodle(22, 22), Miniature Poodle(12, 12), Standard Poodle (10, 10)
**Working dogs (32)**	Doberman Pinscher(6, 6), German Shepherd(10, 10)
**Small Terriers (236)**	Cairn Terrier(4, 4), Jack Russell Terrier(58, 58), West Highland White Terrier(10, 10), Yorkshire Terrier(46, 46)
**Sight hounds and herding dogs (408)**	Italian Greyhound(18, 18), Whippet(6, 6), Borzoi(2, 2), Pembroke Welsh Corgi(48, 48), Australian Shepherd(6, 6), Border Collie(74, 74), Shetland Sheepdog(50, 50)
**Retrievers (304)**	Labrador Retriever(82, 82), Flat-Coated Retriever(8, 8), Golden Retriever(44, 44), Great Dane(2, 2), Bernese Mountain Dog(16, 16)
**Mastiff-like dogs (112)**	Boston Terrier(32, 32), Boxer(4, 4), Bulldog(2, 2), French Bulldog(18, 18)

The numbers of selected dogs are shown in parentheses.

**Table 3:** Results for the analysis of factor scores using generalized linear models.

	F1: Aggression to unfamiliar persons					F2: Fear of unfamiliar persons					F3: Trainability				
	χ^2^	df	p	Pairwise comparison	B^5)^	χ^2^	df	p	Pairwise comparison	B	χ^2^	df	p	Pairwise comparison	B
Breed groups^1)^	50.570	7	0.000	1 > 7, 2 > 6, 2 > 7, 2 > 8, 3 > 7, 3 > 8, 5 > 7, 6 > 7		126.652	7	0.000	1 > 4, 2 > 4, 3 > 4, 5 > 4, 6 > 4, 7 > 4, 8 > 4		51.531	7	0.000	6 > 1, 6 > 2, 6 > 3, 6 > 5, 6 > 7, 6 > 8	
Country	1.015	1	0.314			70.010	1	0.000	US > JPN		14.488	1	0.000	US > JPN	
Sex	1.683	1	0.195			69.123	1	0.000	F > M		0.749	1	0.387		
Neutered status^2)^	0.658	1	0.417			0.468	1	0.494			3.771	1	0.052		
Source where aquired^3)^	24.100	6	0.001	2 > 3, 5 > 3		1.940	6	0.925			31.588	6	0.000	3 > 4, 7 > 4	
Dog-ownership experience^4)^	1.361	1	0.243			0.006	1	0.939			15.057	1	0.000	2 > 1	
Body weight	0.571	1	0.450			14.518	1	0.000		−0.029	3.725	1	0.054		
Dog’s age at evaluation	0.000	1	0.996			0.627	1	0.429			22.534	1	0.000		0.024
Dog’s age when acquired	14.696	1	0.000		−0.003	3.223	1	0.073			5.055	1	0.025		0.000
Breed groups*Country	61.631	15	0.000			139.576	15	0.000			89.920	15	0.000		
Breed groups*Sex	63.198	15	0.000			127.814	15	0.000			59.386	15	0.000		
Country*Sex	12.030	3	0.007			85.804	3	0.000			15.663	3	0.001		
Breed groups*Country*Sex	31	0.000				141.411	31	0.000			104.545	31	0.000		
Omnibus	189.444	42	0.000			145.060	42	0.000			248.373	42	0.000		
	F4: Separation-related anxiety					F5: Energy and restless					F6: Fear of non-social stimuli				
	χ^2^	df	p	Pairwise comparison	B	χ^2^	df	p	Pairwise comparison	B	χ^2^	df	p	Pairwise comparison	B
Breed groups^1)^	13.498	7	0.061			44.844	7	0.000	1 > 4, 2 > 4, 3 > 4, 5 > 4, 6 > 4, 7 > 4, 6 > 3, 6 > 8		44.844	7	0.000	1 > 4, 2 > 4, 3 > 4, 6 > 3, 5 > 4, 6 > 4, 7 > 4, 6 > 8	
Country	31.078	1	0.000	US > JPN		5.077	1	0.024	JPN > US		5.077	1	0.024	JPN > US	
Sex	0.000	1	0.996			0.161	1	0.688			0.161	1	0.688		
Neutered status^2)^	4.368	1	0.037	I > N		5.631	1	0.018	N > I		5.631	1	0.018	N > I	
Source where aquired^3)^	12.795	6	0.046			10.706	6	0.098			10.706	6	0.098		
Dog-ownership experience^4)^	10.161	1	0.001	1 > 2		2.130	1	0.144			2.130	1	0.144		
Body weight	4.925	1	0.026		−0.012	7.906	1	0.005		−0.012	7.906	1	0.005		−0.012
Dog’s age at evaluation	5.444	1	0.020		−0.039	2.154	1	0.142			2.154	1	0.142		
Dog’s age when acquired	1.083	1	0.298			4.155	1	0.042		0.001	4.155	1	0.042		0.001
Breed groups*Country	96.176	15	0.000			52.434	15	0.000			52.434	15	0.000		
Breed groups*Sex	20.717	15	0.146			47.984	15	0.000			47.984	15	0.000		
Country*Sex	31.078	3	0.000			5.752	3	0.124			5.752	3	0.124		
Breed groups*Country*Sex	106.105	31	0.000			65.671	31	0.000			65.671	31	0.000		
Omnibus	237.075	42	0.000			148.076	42	0.000			148.076	42	0.000		
															
	F7: Aggression to household members					F8: Fear of unfamiliar dogs					F9: Aggression to unfamiliar dogs				
	χ2	df	p	Pairwise comparison	B	χ^2^	df	p	Pairwise comparison	B	χ^2^	df	p	Pairwise comparison	B
Breed groups^1)^	84.719	7	0.000	1 > 4, 2 > 4, 2 > 6, 2 > 7, 3 > 4, 3 > 6, 5 > 4, 6 > 4, 7 > 4, 8 > 4		141.880	7	0.000	1 > 4, 2 > 4, 3 > 4, 5 > 4, 6 > 4, 7 > 4, 8 > 4		26.211	7	0.000	1 > 7, 2 > 7, 5 > 7, 6 > 7	
Country	0.000	1	1.000			80.139	1	0.000	US > JPN		2.747	1	0.097		
Sex	0.000	1	1.000			78.662	1	0.000	F > M		5.105	1	0.024	M > F	
Neutered status^2)^	0.000	1	1.000			2.798	1	0.094			0.509	1	0.476		
Source where aquired^3)^	0.000	6	1.000			11.911	6	0.064			11.427	6	0.076		
Dog-ownership experience^4)^	0.000	1	1.000			1.017	1	0.313			0.780	1	0.377		
Body weight	0.023	1	0.881			5.361	1	0.021		−0.017	1.718	1	0.190		
Dog’s age at evaluation	0.000	1	0.986			1.257	1	0.262			16.415	1	0.000		0.069
Dog’s age when acquired	2.897	1	0.089			0.008	1	0.929			2.756	1	0.097		
Breed groups*Country	89.609	14	0.000			153.605	15	0.000			36.747	15	0.001		
Breed groups*Sex	85.619	14	0.000			142.858	15	0.000			38.165	15	0.001		
Country*Sex	20.113	3	0.000			98.852	3	0.000			9.449	3	0.024		
Breed groups*Country*Sex	91.284	29	0.000			155.422	31	0.000			52.726	31	0.009		
Omnibus	266.838	42	0.000			111.849	42	0.000			104.112	42	0.000		
	F11: Attachment and attention-seeking					F12: Aggression to persons passing near the house									
	χ^2^	df	p	Pairwise comparison	B	χ^2^	df	p	Pairwise comparison	B					
Breed groups^1)^	82.116	7	0.000	2 > 1, 3 > 1, 4 > 1, 5 > 1, 6 > 1, 7 > 1, 8 > 1		19.286	7	0.007	2 > 8						
Country	136.912	1	0.000	US > JPN		3.724	1	0.054							
Sex	3.030	1	0.082			0.984	1	0.321							
Neutered status^2)^	1.019	1	0.313			0.543	1	0.461							
Source where aquired^3)^	11.958	6	0.063			8.539	6	0.201							
Dog-ownership experience^4)^	0.042	1	0.837			0.335	1	0.563							
Body weight	7.023	1	0.008		−0.005	0.007	1	0.934							
Dog’s age at evaluation	11.145	1	0.001		−0.018	0.000	1	0.999							
Dog’s age when acquired	1.318	1	0.251			8.065	1	0.005		−0.002					
Breed groups*Country	328.227	15	0.000			29.034	15	0.016							
Breed groups*Sex	86.349	15	0.000			26.774	15	0.031							
Country*Sex	145.751	3	0.000			7.556	3	0.056							
Breed groups*Country*Sex	344.926	31	0.000			47.057	31	0.032							
Omnibus	385.296	42	0.000			72.384	42	0.002							

1) Ancient and spitz breeds: 1, Toy dogs: 2, Spaniels, scent hounds, and poodles: 3, Working dogs: 4, Small terriers: 5, Sight hounds and herding dogs: 6, Retrievers: 7, Mastiff-like dogs: 8.

2) neutered: N, intact: I.

3) bred by owner: 1, friend of relative: 2, breeder: 3, pet store: 4, shelter: 5, stray: 6, other: 7.

4) first ownership: 1, second and more ownership: 2.

5) partial regression coefficient”.
