# Supplementary material for: Comparison of owner-reported behavioral characteristics among genetically clustered breeds of dog (Canis familiaris)
Source: Sci Rep. 2015 Dec 18;5:17710. doi: 10.1038/srep17710 (PMC4683527; doi:10.1038/srep17710)
Supplement: Supplementary Figure 3 [file srep17710-s3.pdf]

Supplementary Figure 3. The Breed Tree of the Cladistic Analysis for Factor 2 (Fear of unfamiliar persons).

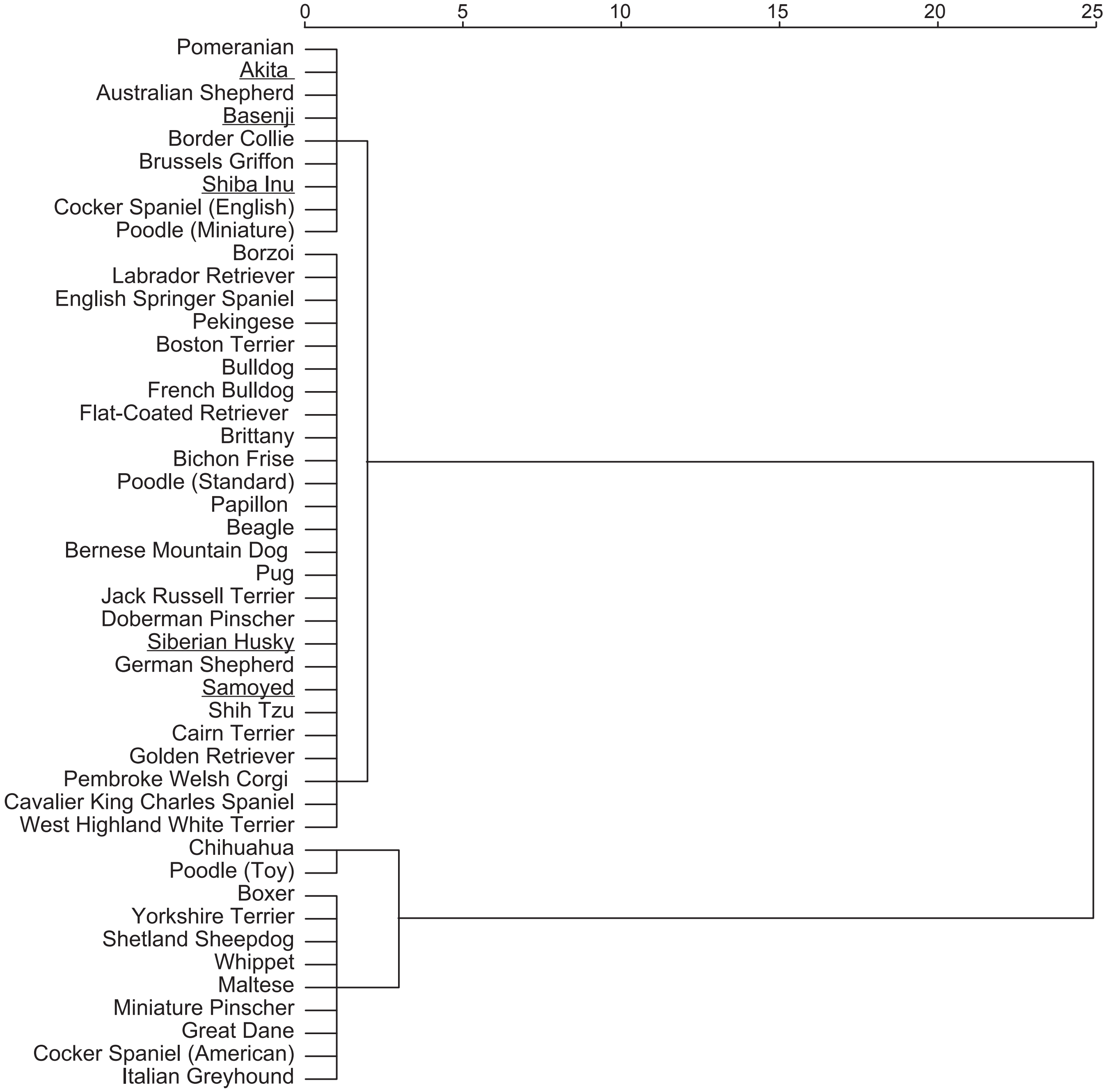

The breeds of the Ancient and spitz breed group are underlined.
