# Supplementary material for: Comparison of owner-reported behavioral characteristics among genetically clustered breeds of dog (Canis familiaris)
Source: Sci Rep. 2015 Dec 18;5:17710. doi: 10.1038/srep17710 (PMC4683527; doi:10.1038/srep17710)
Supplement: Supplementary Table 1 [file srep17710-s4.pdf]

Comparison of owner-reported behavioral characteristics among genetically clustered breeds of dog (Canis familiaris).
Akiko Tonoike, Miho Nagasawa, Kazutaka Mogi, James A. Serpell, Hisashi Ohtsuki, Takefumi Kikusui

Supplementary Table 1 The Number of each breed and sex in Japan and the United States

a. Japan

Breed	Male (intact)	Male (castrated)	Female (intact)	Female (spayed)	Total	Cumulative %
**Poodle (Toy)**	74 (2)	135 (9)	57 (3)	121 (8)	387	13.11
**Dachshund (Miniature)**	80 (11)	85 (10)	49 (8)	93 (13)	307	23.52
**Shiba Inu**	58 (16)	49 (16)	35 (6)	98 (26)	240	31.65
**Chihuahua**	32 (15)	69 (40)	34 (25)	55 (30)	190	38.09
**Labrador Retriever**	13 (12)	30 (28)	15 (14)	30 (27)	88	41.07
**Border Collie**	20 (19)	23 (19)	14 (13)	27 (24)	84	43.92
**Papillon**	12 (4)	23 (8)	13 (3)	36 (9)	84	46.76
**Miniature Schnauzer**	15 (10)	29 (20)	17 (13)	20 (17)	81	49.51
**Pembroke Welsh Corgi**	18 (10)	21 (14)	11 (7)	27 (17)	77	52.12
**French Bulldog**	18 (4)	21 (5)	7 (2)	25 (7)	71	54.52
**Jack Russell Terrier**	15 (11)	25 (18)	6 (6)	23 (23)	69	56.86
**Pomeranian**	4 (3)	33 (22)	11 (11)	16 (14)	64	59.03
**Yorkshire Terrier**	16 (10)	20 (13)	10 (10)	15 (13)	61	61.10
**Shetland Sheepdog**	12 (12)	14 (13)	16 (11)	16 (14)	58	63.06
**Golden Retriever**	13 (5)	18 (17)	6 (6)	16 (16)	53	64.86
**Pug**	13 (13)	10 (10)	7 (7)	18 (16)	48	66.49
**Cavalier King Charles Spaniel**	6 (6)	13 (12)	4 (2)	23 (16)	46	68.04
**Beagle**	7 (7)	14 (14)	6 (5)	16 (16)	43	69.50
**Maltese**	7 (4)	20 (12)	8 (8)	8 (8)	43	70.96
**Shih Tzu**	8 (8)	13 (12)	8 (7)	14 (13)	43	72.42
**Italian Greyhound**	8 (1)	16 (8)	3 (2)	11 (7)	38	73.70
**Boston Terrier**	9 (8)	9 (8)	2 (2)	15 (14)	35	74.89
**Miniature Pinscher**	8 (7)	9 (6)	4 (3)	10 (10)	31	75.94
**Cocker Spaniel (American)**	4 (3)	8 (4)	1 (1)	6 (6)	19	76.58
**Bernese Mountain Dog**	8 (7)	2 (1)	4 (4)	4 (4)	18	77.19
**Flat-Coated Retriever**	2 (2)	2 (2)	3 (1)	9 (3)	16	77.74
**Poodle (Miniature)**	4 (2)	4 (4)	3 (2)	5 (4)	16	78.28
**German Shepherd**	5 (4)	1 (1)	4 (3)	4 (2)	14	78.75
**Bichon Frise**	1 (1)	5 (4)	1 (1)	5 (4)	12	79.16
**Poodle (Standard)**	2 (1)	4 (4)	3 (2)	3 (3)	12	79.57
**Cocker Spaniel (English)**	2 (2)	3 (3)	2 (2)	4 (3)	11	79.94
**West Highland White Terrier**	2 (1)	4 (4)	2 (2)	3 (3)	11	80.31
Japanese Spitz	2	1	2	5	10	80.65
Kai	4	2	1	3	10	80.99
**Pekingese**	2 (1)	4 (3)	3 (3)	1 (1)	10	81.33
**Akita**	3 (2)	2	1 (1)	2 (1)	8	81.60
**Australian Shepherd**	2 (1)	3 (2)	0	3 (3)	8	81.87
**Doberman Pinscher**	2	3 (3)	0	3 (3)	8	82.14
**Siberian Husky**	5 (1)	2	1 (1)	0	8	82.41
**Dalmatian**	1 (1)	2 (2)	1 (1)	3 (2)	7	82.65
**Whippet**	2 (2)	2 (1)	0	3 (3)	7	82.89
Miniature Bull Terrier	1	2	0	3	6	83.09
**Pointer**	1 (1)	3 (1)	1 (1)	1 (1)	6	83.29
**Basenji**	2 (2)	0	0	3 (2)	5	83.46
**Belgian Groenendal**	1	3 (1)	0	1 (1)	5	83.63
**Boxer**	1 (1)	1 (1)	0	3 (2)	5	83.80
**Cairn Terrier**	1 (1)	1 (1)	1 (1)	2 (1)	5	83.97
Cardigan Welsh Corgi	0	1	2	2	5	84.14
**English Setter**	1 (1)	3	0	1 (1)	5	84.31
**Fox Terrier (Wire/Wire-haired)**	2 (1)	0	0	3 (1)	5	84.48
**Airedale Terrier**	2 (1)	1	0	1 (1)	4	84.62
**Border Terrier**	1	2 (1)	0	1 (1)	4	84.75
**English Springer Spaniel**	1 (1)	1 (1)	0	2 (2)	4	84.89
Hokkaido Inu	1	1	0	2	4	85.02
Irish Setter	1	0	1	2	4	85.16
Kishu Inu	0	2	2	0	4	85.29
Kooikwehondje	1	3	0	0	4	85.43
Leonberger	2	0	0	2	4	85.56
Norfolk Terrier	0	1	0	3	4	85.70
**Samoyed**	0	1 (1)	1	2 (1)	4	85.84
White Swiss Shepherd Dog	3	0	1	0	4	85.97
**Belgian Tervuren**	0	1 (1)	1 (1)	1	3	86.07
Bolognese	0	1	1	1	3	86.17
**Borzoi**	1 (1)	0	1	1 (1)	3	86.28
**Brussels Griffon**	2 (1)	0	0	1 (1)	3	86.38
**Bulldog**	1	1 (1)	0	1 (1)	3	86.48
Collie (Rough)	0	0	2	1	3	86.58
Japanese Chin	0	3	0	0	3	86.68
**Manchester Terrier (toy)**	1 (1)	0	1 (1)	1	3	86.78

Breed	Male (intact)	Male (castrated)	Female (intact)	Female (spayed)	Total	Cumulative %
Old English Sheepdog	2	1	0	0	3	86.89
**Australian Kelpie**	0	1 (1)	0	1 (1)	2	86.95
**Brittany**	0	1 (1)	0	1 (1)	2	87.02
Dachshund	1	1	0	0	2	87.09
**Great Dane**	0	1 (1)	0	1 (1)	2	87.16
Great Pyrenees	0	0	0	2	2	87.22
**Lakeland Terrier**	1 (1)	0	1 (1)	0	2	87.29
Norwich Terrier	0	2	0	0	2	87.36
Petit Basset Griffon Vendeen	0	0	0	2	2	87.43
Scottish Terrier	0	0	1	1	2	87.50
Tibetan Spaniel	2	0	0	0	2	87.56
Weimaraner	1	1	0	0	2	87.63
Afghan Hound	0	0	1	0	1	87.67
American Terrier	0	1	0	0	1	87.70
Basset Hound	0	0	0	1	1	87.73
Bearded Collie	0	0	0	1	1	87.77
Belgian Griffon	0	1	0	0	1	87.80
Belgian Malinois	1	0	0	0	1	87.83
Bouvier des Flandres	0	0	0	1	1	87.87
Bull Terrier	1	0	0	0	1	87.90
Chinese Crested	1	0	0	0	1	87.94
Collie (Smooth)	0	0	1	0	1	87.97
Dogo Argentino	0	0	1	0	1	88.00
Fox Terrier	0	0	0	1	1	88.04
German Shorthaired Pointer	0	0	1	0	1	88.07
Havanese	0	0	1	0	1	88.11
Iceland Sheepdog	0	1	0	0	1	88.14
Japanese Terrier	0	0	1	0	1	88.17
Kawakami Inu	0	0	1	0	1	88.21
Labradoodle	0	1	0	0	1	88.24
Lhasa Apso	0	0	1	0	1	88.28
Maremma Sheepdog	0	0	0	1	1	88.31
New Zealand Heading Dog	0	0	0	1	1	88.34
Petit Brabancon	0	0	1	0	1	88.38
Plott Hound	1	0	0	0	1	88.41
Polish Lowland Sheepdog	1	0	0	0	1	88.44
Poodlepointer	0	1	0	0	1	88.48
Rottweiler	0	1	0	0	1	88.51
Saluki	0	1	0	0	1	88.55
Schipperke	0	0	0	1	1	88.58
Shikoku Inu	1	0	0	0	1	88.61
Spanish Water Dog	0	1	0	0	1	88.65
Tibetan Mastiff	1	0	0	0	1	88.68
Toy Fox Terrier	0	1	0	0	1	88.72
Vizsla	0	1	0	0	1	88.75
White Shepherd Dog	0	0	0	1	1	88.78
Mixed Breed/Unknown	48	97	50	136	331	100.00
**Total**	**608 (242)**	**900 (384)**	**451 (203)**	**992 (423)**	**2951**	

b. The United States

Breed	Male (intact)	Male (castrated)	Female (intact)	Female (spayed)	Total	Cumulative %
**Labrador Retriever**	52 (5)	267 (36)	40 (6)	259 (35)	618	5.95
**German Shepherd**	69	149 (5)	65	181 (5)	464	10.41
**Golden Retriever**	42 (5)	132 (17)	39 (8)	120 (14)	333	13.62
**Border Collie**	34 (6)	105 (31)	31 (8)	99 (29)	269	16.21
Rottweiler	51	85	50	79	265	18.76
**Australian Shepherd**	27 (1)	109 (2)	26 (1)	96 (2)	258	21.24
**Doberman Pinscher**	49 (1)	57 (2)	38 (1)	69 (2)	213	23.29
Soft Coated Wheaten Terrier	28	63	30	67	188	25.10
American Pit Bull Terrier	11	87	10	73	181	26.85
**Poodle (Standard)**	21 (2)	70 (4)	15 (1)	57 (3)	163	28.41
Australian Cattle Dog	13	69	10	69	161	29.96
Collie	35	38	30	32	135	31.26
Mastiff (English)	32	52	24	26	134	32.55
**Boxer**	11 0	53 (2)	12 (1)	55 (1)	131	33.81
**Bernese Mountain Dog**	17 (7)	49 (1)	25 (2)	34 (6)	125	35.02
**Cocker Spaniel (American)**	11 (4)	58 (3)	13 (2)	43 (5)	125	36.22
**Jack Russell Terrier**	16 (6)	58 (23)	11 (5)	35 (24)	120	37.38
**Chihuahua**	5 (5)	50 (50)	11 (11)	45 (44)	111	38.44
**Akita**	27 (1)	34 (1)	15 (1)	31 (1)	107	39.47
**Great Dane**	9	40 (1)	12	40 (1)	101	40.45
**Beagle**	6 (4)	45 (17)	6 (5)	41 (16)	98	41.39
**Siberian Husky**	15 (1)	37	12	28 (1)	92	42.28
**Shetland Sheepdog**	7 (4)	44 (21)	9 (5)	31 (20)	91	43.15

Breed	Male (intact)	Male (castrated)	Female (intact)	Female (spayed)	Total	Cumulative %
Havanese	11	35	21	20	87	43.99
Portuguese Water Dog	13	23	24	26	86	44.82
Rhodesian Ridgeback	24	22	22	15	83	45.62
**Bichon Frise**	3	35 (5)	2	42 (5)	82	46.40
**Shiba Inu**	12 (7)	38 (25)	10 (10)	22 (22)	82	47.19
Dachshund	7	40	9	19	75	47.92
**English Springer Spaniel**	13 (1)	29 (1)	10 (1)	22 (1)	74	48.63
**Pomeranian**	16 (8)	28 (17)	8 (8)	19 (17)	71	49.31
**Airedale Terrier**	10 (1)	22	8	29 (1)	69	49.98
Belgian Malinois	15	17	14	23	69	50.64
Greyhound	0	31	0	37	68	51.29
**Shih Tzu**	5 (2)	24 (18)	6 (5)	28 (15)	63	51.90
**Maltese**	4 (4)	26 (12)	5 (3)	27 (13)	62	52.50
**Miniature Schnauzer**	4 (3)	28 (27)	5 (5)	25 (25)	62	53.09
**Belgian Tervuren**	22 (1)	11	17	8 (1)	58	53.65
**Cocker Spaniel (English)**	18 (3)	10 (2)	18 (2)	12 (3)	58	54.21
**Pug**	2 (2)	28 (21)	2 (1)	25 (22)	57	54.76
Weimaraner	8	22	9	18	57	55.31
**Whippet**	11 (1)	20 (2)	9 (1)	15 (2)	55	55.84
**Brittany**	2	28 (1)	4 (1)	19	53	56.35
Rat Terrier	3	21	10	19	53	56.86
**Pembroke Welsh Corgi**	4 (3)	24 (21)	2 (2)	22 (22)	52	57.36
**Yorkshire Terrier**	4 (4)	22 (19)	6 (4)	20 (19)	52	57.86
Pit Bull	5	19	2	25	51	58.35
American Staffordshire Terrier	5	22	2	21	50	58.83
**Dalmatian**	13 (1)	16 (2)	6 (1)	15 (2)	50	59.31
Chinook	9	7	20	13	49	59.78
**Boston Terrier**	3	27 (16)	1 (1)	16 (15)	47	60.24
**Dachshund (Miniature)**	4 (3)	20 (18)	2 (2)	19 (19)	45	60.67
**Cavalier King Charles Spaniel**	5 (2)	19 (16)	5 (4)	15 (14)	44	61.09
**English Setter**	13 (1)	11	10 (1)	6	40	61.48
**West Highland White Terrier**	5 0	20 (5)	1	14 (5)	40	61.86
German Shorthaired Pointer	9	12	2	16	39	62.24
Eurasier	11	5	10	12	38	62.60
Vizsla	9	14	7	8	38	62.97
**Papillon**	7 (3)	18 (9)	6 (6)	6 (6)	37	63.33
**Poodle (Miniature)**	2	17 (6)	3 (2)	15 (4)	37	63.68
Basset Hound	3	19	1	12	35	64.02
Bullmastiff	6	9	7	12	34	64.35
**Cairn Terrier**	0	22 (2)	0	11 (2)	33	64.66
Great Pyrenees	3	19	0	11	33	64.98
American Eskimo Dog	0	19	1	12	32	65.29
Belgian Sheepdog	7	10	8	6	31	65.59
Irish Setter	18	3	9	1	31	65.89
**Miniature Pinscher**	4 (3)	14 (10)	4 (4)	9 (9)	31	66.19
Saint Bernard	5	13	4	9	31	66.48
Alaskan Malamute	6	11	0	12	29	66.76
Lhasa Apso	1	12	0	16	29	67.04
Irish Wolfhound	13	9	3	3	28	67.31
**Flat-Coated Retriever**	3 (2)	7 (2)	8 (3)	9 (1)	27	67.57
Alaskan Husky	6	8	3	9	26	67.82
Chesapeake Bay Retriever	7	6	2	11	26	68.07
**Poodle (Toy)**	3 (2)	12 (9)	1 (1)	10 (10)	26	68.32
Redbone Coonhound	1	11	1	13	26	68.57
Staffordshire Bull Terrier	2	5	2	17	26	68.82
American Bulldog	3	14	1	7	25	69.06
**Border Terrier**	5	9 (1)	4	7 (1)	25	69.30
Bouvier des Flandres	7	8	3	7	25	69.54
Nova Scotia Duck Tolling Retriever	6	7	5	6	24	69.78
Chinese Shar-Pei	3	10	1	9	23	70.00
**Australian Kelpie**	6 (1)	5	1	10 (1)	22	70.21
**Borzoi**	10 (1)	3	6 (1)	2	21	70.41
**Bulldog**	3 (1)	4	4 (1)	10	21	70.61
Newfoundland	2	7	4	8	21	70.82
Afghan Hound	6	1	9	3	19	71.00
**Fox Terrier (Wire/Wire-haired)**	1	10 (1)	2	5 (1)	18	71.17
**French Bulldog**	0	9 (9)	2 (2)	7 (7)	18	71.34
**Italian Greyhound**	3 (3)	6 (6)	3 (3)	6 (6)	18	71.52
Treeing Walker Coonhound	1	10	0	7	18	71.69
Black and Tan Coonhound	2	5	3	7	17	71.85
Chinese Crested	3	7	2	5	17	72.02
English Bulldog	2	6	0	9	17	72.18
Norwegian Elkhound	2	5	1	9	17	72.35
**Basenji**	2 (1)	8 (1)	1 (1)	5 (1)	16	72.50
German Wirehaired Pointer	2	5	3	5	15	72.64

Breed	Male (intact)	Male (castrated)	Female (intact)	Female (spayed)	Total	Cumulative %
Kuvasz	4	5	5	1	15	72.79
**Pekingese**	1 (1)	8 (3)	0	6 (4)	15	72.93
Chow Chow	6	2	2	4	14	73.07
Scottish Terrier	4	6	2	2	14	73.20
Anatolian Shepherd	0	8	1	4	13	73.33
Cane Corso	1	2	3	7	13	73.45
Cardigan Welsh Corgi	2	5	2	4	13	73.58
Gordon Setter	5	2	6	0	13	73.70
Husky Mix	1	7	0	5	13	73.83
Parson Russell Terrier	1	7	2	3	13	73.95
**Pointer**	2	6 (2)	1	4 (2)	13	74.08
Schipperke	2	5	2	4	13	74.20
Bearded Collie	2	6	1	3	12	74.32
Collie (Rough)	2	5	1	4	12	74.43
Giant Schnauzer	2	3	2	5	12	74.55
**Samoyed**	1	3 (1)	3 (1)	5	12	74.67
Tibetan Terrier	2	3	4	3	12	74.78
Lagotto Romagnolo	5	0	4	2	11	74.89
Old English Sheepdog	0	9	0	2	11	74.99
Plott	0	7	0	4	11	75.10
American Foxhound	0	1	0	9	10	75.19
Fox Terrier (Toy)	0	4	1	5	10	75.29
Ibizan Hound	4	1	2	3	10	75.39
Irish Water Spaniel	5	1	4	0	10	75.48
Keeshond	2	5	0	3	10	75.58
Silky Terrier	0	4	2	4	10	75.68
Welsh Terrier	0	6	2	2	10	75.77
Collie (Smooth)	0	5	1	3	9	75.86
Blue Heeler	1	2	1	4	8	75.94
Bull Terrier	0	5	1	2	8	76.01
Fox Terrier (Smooth)	2	3	0	3	8	76.09
Standard Schnauzer	1	4	0	3	8	76.17
Blue Tick Coonhound	0	5	1	1	7	76.23
**Brussels Griffon**	0	4 (1)	1 (1)	2	7	76.30
Lurcher	1	4	0	2	7	76.37
Black Russian Terrier	2	2	0	2	6	76.43
Irish Terrier	3	1	0	2	6	76.48
Jindo	1	3	0	2	6	76.54
Kerry Blue Terrier	2	2	1	1	6	76.60
Norwich Terrier	0	3	2	1	6	76.66
Spanish Water Dog	2	1	1	2	6	76.72
Sussex Spaniel	1	3	2	0	6	76.77
Welsh Springer Spaniel	2	3	1	0	6	76.83
American Boxer	1	1	0	3	5	76.88
Australian Silky Terrier	0	4	0	1	5	76.93
Australian Terrier	1	3	0	1	5	76.98
Briard	0	1	3	1	5	77.02
Greater Swiss Mountain Dog	0	2	1	2	5	77.07
Irish Red and White Setter	3	0	1	1	5	77.12
Neapolitan Mastiff	0	2	0	3	5	77.17
Polish Lowland Sheepdog	1	4	0	0	5	77.22
Saluki	2	2	1	0	5	77.26
Affenpinscher	0	3	0	1	4	77.30
Clumber Spaniel	1	3	0	0	4	77.34
Eurohound Husky	1	1	2	0	4	77.38
Japanese Chin	0	1	0	3	4	77.42
**Manchester Terrier (Toy)**	0	1 (1)	0	3 (1)	4	77.46
Pharaoh Hound	1	2	0	1	4	77.50
**Belgian Groenendal**	1	1 (1)	0	1 (1)	3	77.52
Dogo Argentino	1	1	0	1	3	77.55
Lowchen	1	2	0	0	3	77.58
Manchester Terrier	0	1	0	2	3	77.61
Miniature Bull Terrier	1	0	0	2	3	77.64
Shiloh Shepherd	0	0	0	3	3	77.67
WGSD	0	2	1	0	3	77.70
American Water Spaniel	0	0	1	1	2	77.72
Bedlington Terrier	1	0	1	0	2	77.74
Bracco Italiano	0	0	0	2	2	77.76
Curly-Coated Retriever	2	0	0	0	2	77.77
English Foxhound	0	1	0	1	2	77.79
Field Spaniel	1	0	0	1	2	77.81
King Shepherd	0	2	0	0	2	77.83
**Lakeland Terrier**	0	1 (1)	1 (1)	0	2	77.85
Mudi	0	0	2	0	2	77.87
Petit Basset Griffon Vendeen	1	1	0	0	2	77.89

Breed	Male (intact)	Male (castrated)	Female (intact)	Female (spayed)	Total	Cumulative %
Scottish Deerhound	0	0	1	1	2	77.91
Silken Windhound	1	0	1	0	2	77.93
Spinone Italiano	1	1	0	0	2	77.95
Stabyhoun	0	0	2	0	2	77.97
Tibetan Spaniel	0	0	1	1	2	77.99
Wirehaired Pointing Griffon	0	0	0	2	2	78.01
Beauceron	0	0	1	0	1	78.02
Bloodhound	0	0	0	1	1	78.02
English Coonhound	0	0	0	1	1	78.03
German Pinscher	0	0	1	0	1	78.04
Icelandic Sheepdog	0	1	0	0	1	78.05
Komondor	1	0	0	0	1	78.06
Lancashire Heeler	1	0	0	0	1	78.07
Otterhound	1	0	0	0	1	78.08
Peruvian Inca Orchid	0	0	0	1	1	78.09
Portuguese Podengo	1	0	0	0	1	78.10
Puli	0	0	1	0	1	78.11
Sloughi	1	0	0	0	1	78.12
Swiss Mountain Dog	0	0	1	0	1	78.13
Teddy Roosevelt Terriers	0	0	1	0	1	78.14
Tosa	0	1	0	0	1	78.15
Mixed Breed/Unknown	71	1077	55	1067	2270	100.00
**Total**	**1226 (117)**	**4203 (510)**	**1084 (136)**	**3876 (489)**	**10389**	

The breeds written in bold letters were selected for the factor analysis. The numbers of selected dogs are shown in parentheses.
The shaded regions indicated the breeds accounted for 50% of all dogs in each country.
