# Supplementary material for: Comparison of owner-reported behavioral characteristics among genetically clustered breeds of dog (Canis familiaris)
Source: Sci Rep. 2015 Dec 18;5:17710. doi: 10.1038/srep17710 (PMC4683527; doi:10.1038/srep17710)
Supplement: Supplementary Table 2 [file srep17710-s5.pdf]

Comparison of owner-reported behavioral characteristics among genetically clustered breeds of dog (Canis familiaris)

Akiko Tonoike, Miho Nagasawa, Kazutaka Mogi, James A. Serpell, Hisashi Ohtsuki, Takefumi Kikusui

Supplementary Table 2 The results for analysis of factor scores using generalized linear models.

**F1: Aggression to unfamiliar persons**

| | JPN | | | | | US | | | | |
|---|---|---|---|---|---|---|---|---|---|---|
| | χ2 | df | p | Pairwise comparison | B[5)] | χ2 | df | p | Pairwise comparison | B[5)] |
| Breed groups[1)] | 35.683 | 7 | 0.000 | 2>7, 2>8, 3>8 | | 22.108 | 7 | 0.015 | 2>7, 3>7 | |
| Sex | 9.500 | 1 | 0.002 | 1>2 | | 2.006 | 1 | 0.157 | | |
| Neutered status[2)] | 0.540 | 1 | 0.462 | | | 0.034 | 1 | 0.854 | | |
| Source where aquired[3)] | 16.956 | 6 | 0.009 | | | 14.593 | 6 | 0.024 | | |
| Dog-ownership experience[4)] | 0.330 | 1 | 0.566 | | | 2.577 | 1 | 0.108 | | |
| Body weight | 0.499 | 1 | 0.480 | | | 0.251 | 1 | 0.616 | | |
| Dog's age at evaluation | 2.654 | 1 | 0.103 | | | 2.222 | 1 | 0.136 | | |
| Dog's age when acquired | 5.244 | 1 | 0.022 | | -0.004 | 7.269 | 1 | 0.007 | | 0.007 |
| Breed groups*Sex | 9.753 | 7 | 0.203 | | | 7.399 | 7 | 0.389 | | |
| Omnibus | 106.055 | 26 | 0.000 | | | 85.286 | 26 | 0.000 | | |

**F2: Fear of unfamiliar persons**

| | JPN | | | | | US | | | | |
|---|---|---|---|---|---|---|---|---|---|---|
| | χ2 | df | p | Pairwise comparison | B | χ2 | df | p | Pairwise comparison | B |
| Breed groups[1)] | 6.002 | 7 | 0.540 | | | 11.446 | 7 | 0.120 | | |
| Sex | 10.172 | 1 | 0.001 | 1<2 | | 0.923 | 1 | 0.337 | | |
| Neutered status[2)] | 0.004 | 1 | 0.948 | | | 1.303 | 1 | 0.254 | | |
| Source where aquired[3)] | 2.180 | 6 | 0.902 | | | 1.533 | 6 | 0.957 | | |
| Dog-ownership experience[4)] | 0.784 | 1 | 0.376 | | | 0.054 | 1 | 0.816 | | |
| Body weight | 5.620 | 1 | 0.018 | | -0.036 | 7.809 | 1 | 0.005 | | -0.024 |
| Dog's age at evaluation | 0.696 | 1 | 0.404 | | | 4.743 | 1 | 0.029 | | -0.059 |
| Dog's age when acquired | 0.020 | 1 | 0.887 | | | 5.806 | 1 | 0.016 | | 0.002 |
| Breed groups*Sex | 6.224 | 6 | 0.399 | | | 3.421 | 7 | 0.843 | | |
| Omnibus | 46.087 | 26 | 0.009 | | | 83.530 | 26 | 0.000 | | |

**F3: Trainability**

| | JPN | | | | | US | | | | |
|---|---|---|---|---|---|---|---|---|---|---|
| | χ2 | df | p | Pairwise comparison | B | χ2 | df | p | Pairwise comparison | B |
| Breed groups[1)] | 36.607 | 7 | 0.000 | 1<6, 3<6, 5<6 | | 20.077 | 7 | 0.005 | 1<6, 5<6 | |
| Sex | 0.566 | 1 | 0.452 | | | 0.067 | 1 | 0.796 | | |
| Neutered status[2)] | 6.678 | 1 | 0.010 | 1>2 | | 0.682 | 1 | 0.409 | | |
| Source where aquired[3)] | 15.233 | 6 | 0.019 | 3>4 | | 15.166 | 6 | 0.019 | 4>7 | |
| Dog-ownership experience[4)] | 4.569 | 1 | 0.033 | 1<2 | | 14.238 | 1 | 0.000 | 1<2 | |
| Body weight | 1.080 | 1 | 0.299 | | | 3.634 | 1 | 0.057 | | |
| Dog's age at evaluation | 13.250 | 1 | 0.000 | | 0.034 | 13.008 | 1 | 0.000 | | 0.019 |
| Dog's age when acquired | 0.342 | 1 | 0.559 | | | 7.406 | 1 | 0.006 | | -0.001 |
| Breed groups*Sex | 6.006 | 7 | 0.539 | | | 2.581 | 7 | 0.921 | | |
| Omnibus | 102.518 | 26 | 0.000 | | | 117.931 | 26 | 0.000 | | |

**F4: Separation-related anxiety**

| | JPN | | | | | US | | | | |
|---|---|---|---|---|---|---|---|---|---|---|
| | χ2 | df | p | Pairwise comparison | B[5)] | χ2 | df | p | Pairwise comparison | B[5)] |
| Breed groups[1)] | 7.479 | 7 | 0.381 | | | 12.672 | 7 | 0.081 | | |
| Sex | 0.277 | 1 | 0.599 | | | 0.020 | 1 | 0.888 | | |
| Neutered status[2)] | 12.346 | 1 | 0.000 | 1<2 | | 0.091 | 1 | 0.763 | | |
| Source where aquired[3)] | 12.936 | 6 | 0.044 | | | 9.287 | 6 | 0.158 | | |
| Dog-ownership experience[4)] | 1.074 | 1 | 0.300 | | | 12.463 | 1 | 0.000 | 1>2 | |
| Body weight | 0.831 | 1 | 0.362 | | | 3.869 | 1 | 0.049 | | -0.013 |
| Dog's age at evaluation | 1.895 | 1 | 0.169 | | | 5.225 | 1 | 0.022 | | -0.048 |
| Dog's age when acquired | 0.200 | 1 | 0.654 | | | 0.616 | 1 | 0.433 | | |
| Breed groups*Sex | 5.917 | 7 | 0.549 | | | 2.020 | 7 | 0.959 | | |
| Omnibus | 51.301 | 26 | 0.002 | | | 83.212 | 26 | 0.000 | | |

**F5: Energy and restless**

| | JPN | | | | | US | | | | |
|---|---|---|---|---|---|---|---|---|---|---|
| | χ2 | df | p | Pairwise comparison | B | χ2 | df | p | Pairwise comparison | B |
| Breed groups[1)] | 18.468 | 7 | 0.010 | 4<6 | | 19.264 | 7 | 0.007 | 1>4, 4<6 | |
| Sex | 0.386 | 1 | 0.534 | | | 0.006 | 1 | 0.937 | | |
| Neutered status[2)] | 1.344 | 1 | 0.246 | | | 4.358 | 1 | 0.037 | 1>2 | |
| Source where aquired[3)] | 4.287 | 6 | 0.638 | | | 6.516 | 6 | 0.368 | | |
| Dog-ownership experience[4)] | 2.869 | 1 | 0.090 | 1<2 | | 5.462 | 1 | 0.019 | 1<2 | |
| Body weight | 1.980 | 1 | 0.159 | | | 6.001 | 1 | 0.014 | | -0.013 |
| Dog's age at evaluation | 2.532 | 1 | 0.112 | | | 0.077 | 1 | 0.782 | | |
| Dog's age when acquired | 0.267 | 1 | 0.605 | | | 7.783 | 1 | 0.005 | | 0.001 |
| Breed groups*Sex | 2.190 | 7 | 0.949 | | | 6.547 | 7 | 0.477 | | |
| Omnibus | 52.994 | 26 | 0.001 | | | 86.367 | 26 | 0.000 | | |

**F6: Fear of non-social stimuli**

| | JPN | | | | | US | | | | |
|---|---|---|---|---|---|---|---|---|---|---|
| | χ2 | df | p | Pairwise comparison | B | χ2 | df | p | Pairwise comparison | B |
| Breed groups[1)] | 18.468 | 7 | 0.010 | 4<6 | | 19.264 | 7 | 0.007 | 1>4, 4<6 | |
| Sex | 0.386 | 1 | 0.534 | | | 0.006 | 1 | 0.937 | | |
| Neutered status[2)] | 1.344 | 1 | 0.246 | | | 4.358 | 1 | 0.037 | 1>2 | |
| Source where aquired[3)] | 4.287 | 6 | 0.638 | | | 6.516 | 6 | 0.368 | | |
| Dog-ownership experience[4)] | 2.869 | 1 | 0.090 | | | 5.462 | 1 | 0.019 | 1>2 | |
| Body weight | 1.980 | 1 | 0.159 | | | 6.001 | 1 | 0.014 | | -0.013 |
| Dog's age at evaluation | 2.532 | 1 | 0.112 | | | 0.077 | 1 | 0.782 | | |
| Dog's age when acquired | 0.267 | 1 | 0.605 | | | 7.783 | 1 | 0.005 | | 0.001 |
| Breed groups*Sex | 2.190 | 7 | 0.949 | | | 6.547 | 7 | 0.477 | | |
| Omnibus | 52.994 | 26 | 0.001 | | | 86.367 | 26 | 0.000 | | |

| | F7: Aggression to household members | | | | | | | | | |
|---|---|---|---|---|---|---|---|---|---|---|
| | JPN | | | | | US | | | | |
| | χ2 | df | p | Pairwise comparison | B[5] | χ2 | df | p | Pairwise comparison | B[5] |
| Breed groups[1] | 26.656 | 7 | 0.000 | 2>4, 3>4, 4<6 | | 12.400 | 6 | 0.054 | | |
| Sex | 4.384 | 1 | 0.036 | 1<2 | | 0.092 | 1 | 0.761 | | |
| Neutered status[2] | 3.107 | 1 | 0.078 | | | 0.084 | 1 | 0.772 | | |
| Source where aquired[3] | 18.959 | 6 | 0.004 | | | 15.458 | 6 | 0.017 | | |
| Dog-ownership experience[4] | 0.046 | 1 | 0.830 | | | 8.267 | 1 | 0.004 | | |
| Body weight | 0.011 | 1 | 0.918 | | | 0.053 | 1 | 0.818 | | |
| Dog's age at evaluation | 0.152 | 1 | 0.697 | | | 0.044 | 1 | 0.833 | | |
| Dog's age when acquired | 0.130 | 1 | 0.718 | | | 2.596 | 1 | 0.107 | | |
| Breed groups*Sex | 4.039 | 6 | 0.671 | | | 11.672 | 6 | 0.070 | | |
| Omnibus | 108.389 | 26 | 0.000 | | | 74.940 | 26 | 0.000 | | |

| | F8: Fear of unfamiliar dogs | | | | | | | | | |
|---|---|---|---|---|---|---|---|---|---|---|
| | JPN | | | | | US | | | | |
| | χ2 | df | p | Pairwise comparison | B | χ2 | df | p | Pairwise comparison | B |
| Breed groups[1] | 9.250 | 7 | 0.235 | | | 5.621 | 7 | 0.585 | | |
| Sex | 14.742 | 1 | 0.000 | 1<2 | | 0.265 | 1 | 0.606 | | |
| Neutered status[2] | 0.527 | 1 | 0.468 | | | 3.157 | 1 | 0.076 | | |
| Source where aquired[3] | 6.152 | 6 | 0.406 | | | 7.615 | 6 | 0.268 | | |
| Dog-ownership experience[4] | 0.314 | 1 | 0.575 | | | 1.955 | 1 | 0.162 | | |
| Body weight | 3.181 | 1 | 0.075 | | | 2.094 | 1 | 0.148 | | |
| Dog's age at evaluation | 0.261 | 1 | 0.609 | | | 1.152 | 1 | 0.283 | | |
| Dog's age when acquired | 0.018 | 1 | 0.895 | | | 0.039 | 1 | 0.843 | | |
| Breed groups*Sex | 3.663 | 6 | 0.722 | | | 4.145 | 7 | 0.763 | | |
| Omnibus | 59.261 | 26 | 0.000 | | | 50.826 | 26 | 0.003 | | |

| | F9: Aggression to unfamiliar dogs | | | | | | | | | |
|---|---|---|---|---|---|---|---|---|---|---|
| | JPN | | | | | US | | | | |
| | χ2 | df | p | Pairwise comparison | B | χ2 | df | p | Pairwise comparison | B |
| Breed groups[1] | 14.222 | 7 | 0.047 | | | 18.066 | 7 | 0.012 | | |
| Sex | 4.083 | 1 | 0.043 | | | 0.717 | 1 | 0.397 | | |
| Neutered status[2] | 1.623 | 1 | 0.203 | | | 0.184 | 1 | 0.668 | | |
| Source where aquired[3] | 8.789 | 6 | 0.186 | | | 9.291 | 6 | 0.158 | | |
| Dog-ownership experience[4] | 1.935 | 1 | 0.164 | | | 3.048 | 1 | 0.081 | | |
| Body weight | 3.593 | 1 | 0.058 | | | 0.008 | 1 | 0.928 | | |
| Dog's age at evaluation | 14.776 | 1 | 0.000 | | 0.106 | 2.985 | 1 | 0.084 | | |
| Dog's age when acquired | 1.898 | 1 | 0.168 | | | 0.655 | 1 | 0.418 | | |
| Breed groups*Sex | 6.497 | 7 | 0.483 | | | 3.209 | 7 | 0.865 | | |
| Omnibus | 60.411 | 26 | 0.000 | | | 54.225 | 26 | 0.001 | | |

| | F11: Attachment and attention-seeking | | | | | | | | | |
|---|---|---|---|---|---|---|---|---|---|---|
| | JPN | | | | | US | | | | |
| | χ2 | df | p | Pairwise comparison | B[5] | χ2 | df | p | Pairwise comparison | B[5] |
| Breed groups[1] | 55.460 | 7 | 0.000 | 1<2, 1<3, 1<5, 1<6, 1<7, 1<8 | | 35.350 | 7 | 0.000 | 1<2, 1<3, 1<5 | |
| Sex | 6.927 | 1 | 0.008 | 1>2 | | 0.302 | 1 | 0.583 | | |
| Neutered status[2] | 2.800 | 1 | 0.094 | | | 0.017 | 1 | 0.895 | | |
| Source where aquired[3] | 10.261 | 6 | 0.114 | | | 8.593 | 6 | 0.198 | | |
| Dog-ownership experience[4] | 0.675 | 1 | 0.411 | | | 0.002 | 1 | 0.966 | | |
| Body weight | 8.240 | 1 | 0.004 | | -0.009 | 1.112 | 1 | 0.292 | | |
| Dog's age at evaluation | 6.427 | 1 | 0.011 | | -0.021 | 6.039 | 1 | 0.014 | | -0.017 |
| Dog's age when acquired | 1.239 | 1 | 0.266 | | | 0.487 | 1 | 0.485 | | |
| Breed groups*Sex | 3.815 | 7 | 0.801 | | | 6.484 | 7 | 0.484 | | |
| Omnibus | 84.440 | 26 | 0.000 | | | 72.306 | 26 | 0.000 | | |

| | F12: Aggression to persons passing near the house | | | | | | | | | |
|---|---|---|---|---|---|---|---|---|---|---|
| | JPN | | | | | US | | | | |
| | χ2 | df | p | Pairwise comparison | B | χ2 | df | p | Pairwise comparison | B |
| Breed groups[1] | 13.540 | 7 | 0.060 | | | 7.043 | 7 | 0.424 | | |
| Sex | 2.484 | 1 | 0.115 | | | 0.321 | 1 | 0.571 | | |
| Neutered status[2] | 0.857 | 1 | 0.354 | | | 0.006 | 1 | 0.940 | | |
| Source where aquired[3] | 4.902 | 6 | 0.556 | | | 6.490 | 6 | 0.371 | | |
| Dog-ownership experience[4] | 0.180 | 1 | 0.671 | | | 0.595 | 1 | 0.441 | | |
| Body weight | 0.316 | 1 | 0.574 | | | 0.136 | 1 | 0.713 | | |
| Dog's age at evaluation | 0.167 | 1 | 0.682 | | | 0.248 | 1 | 0.619 | | |
| Dog's age when acquired | 0.969 | 1 | 0.325 | | | 7.009 | 1 | 0.008 | | -0.002 |
| Breed groups*Sex | 8.211 | 7 | 0.314 | | | 0.693 | 7 | 0.998 | | |
| Omnibus | 37.725 | 26 | 0.064 | | | 35.618 | 26 | 0.099 | | |

1) Ancient and spitz breeds: 1, Toy dogs: 2, Spaniels, scent hounds, and poodles: 3, Working dogs: 4, Small terriers: 5, Sight hounds and herding dogs: 6, Retrievers: 7, Mastiff-like dogs: 8
2) neutered: N, intact: I
3) bred by owner: 1, friend of relative: 2, breeder: 3, pet store: 4, shelter: 5, stray: 6, other: 7
4) first ownership: 1, second and more ownership: 2
5) partial regression coefficient
