# Supplementary material for: Comparison of owner-reported behavioral characteristics among genetically clustered breeds of dog (Canis familiaris)
Source: Sci Rep. 2015 Dec 18;5:17710. doi: 10.1038/srep17710 (PMC4683527; doi:10.1038/srep17710)
Supplement: Supplementary figure legends [file srep17710-s6.pdf]

Comparison of owner-reported behavioral characteristics among genetically clustered breeds of dog (Canis familiaris).

Akiko Tonoike, Miho Nagasawa, Kazutaka Mogi, James A. Serpell, Hisashi Ohtsuki, Takefumi Kikusui

Supplementary figure legends

Supplementary Figure 1. The breed tree of the cladistic analysis for Factor 11 (Attachment and attention-seeking).

Supplementary Figure 2. The breed tree of the cladistic analysis for Factor 4 (Separation-related anxiety).

Supplementary Figure 3. The breed tree of the cladistic analysis for Factor 2 (Fear of unfamiliar persons).
